# Intraoperative Low Alpha Power in the Electroencephalogram Is Associated With Postoperative Subsyndromal Delirium

**DOI:** 10.3389/fnsys.2019.00056

**Published:** 2019-10-18

**Authors:** Rodrigo Gutierrez, Jose I. Egaña, Iván Saez, Fernando Reyes, Constanza Briceño, Mariana Venegas, Isidora Lavado, Antonello Penna

**Affiliations:** ^1^Departamento de Anestesiología y Medicina Perioperatoria, Hospital Clínico, Universidad de Chile, Santiago, Chile; ^2^Centro de Investigación Clínica Avanzada (CICA), Facultad de Medicina, Hospital Clínico, Universidad de Chile, Santiago, Chile; ^3^Departamento de Terapia Ocupacional y Ciencia de la Ocupación, Facultad de Medicina, Universidad de Chile, Santiago, Chile; ^4^Escuela de Medicina, Facultad de Medicina, Universidad de Chile, Santiago, Chile

**Keywords:** delirium, subsyndromal delirium, anesthesia, electroencephalogram, power spectra, alpha oscillations

## Abstract

**Background:**

Postoperative delirium (PD) and subsyndromal delirium (PSSD) are frequent complications in older patients associated with poor long-term outcome. It has been suggested that certain electroencephalogram features may be capable of identifying patients at risk during surgery. Thus, the goal of this study was to characterize intraoperative electroencephalographic markers to identify patients prone to develop PD or PSSD.

**Methods:**

We conducted an exploratory observational study in older patients scheduled for elective major abdominal surgery. Intraoperative 16 channels electroencephalogram was recorded, and PD/PSSD were diagnosed after surgery with the confusion assessment method (CAM). The total power spectra and relative power of alpha band were calculated.

**Results:**

PD was diagnosed in 2 patients (6.7%), and 11 patients (36.7%) developed PSSD. All of them (13 patients, PD/PSSD group) were compared with patients without any alterations in CAM (17 patients, control group). There were no detectable power spectrum differences before anesthesia between both groups of patients. However, PD/PSSD group in comparison with control group had a lower intraoperative absolute alpha power during anesthesia (4.4 ± 3.8 dB vs. 9.6 ± 3.2 dB, *p* = 0.0004) and a lower relative alpha power (0.09 ± 0.06 vs. 0.21 ± 0.08, *p* < 0.0001). These differences were independent of the anesthetic dose. Finally, relative alpha power had a good ability to identify patients with CAM alterations in the ROC analysis (area under the curve 0.90 (CI 0.78-1), *p* < 0.001).

**Discussion:**

In conclusion, a low intraoperative alpha power is a novel electroencephalogram marker to identify patients who will develop alterations in CAM – i.e., with PD or PSSD – after surgery.

## Introduction

Postoperative delirium (PD) is a frequent clinical condition characterized by an acute disturbance in attention and awareness with a fluctuating evolution found in the first five postoperative days (Inouye et al., 2014b; Evered et al., 2018). It is often diagnosed using a validated tool called Confusion Assessment Method (CAM) (Inouye et al., 1990), which evaluates 4 features: (1) an acute onset of changes or fluctuations in the course of mental status, (2) inattention, (3) disorganized thinking, and (4) an altered level of consciousness. For a patient to be diagnosed as PD, he/she has to manifest both, features 1 and 2, plus either feature 3 or 4 (Inouye et al., 1990). However, growing evidence point out that there is a population of patients who do not meet these CAM criteria, but still present CAM alterations in specific domains – such as inattention, altered level of consciousness or disorganized thinking – a condition known as subsyndromal delirium (SSD) (Levkoff et al., 1996; Marcantonio et al., 2002; Shim et al., 2015). Therefore, PD may manifests as a full entity (Inouye et al., 1990; American Psychiatric Association, 2013; Mashour et al., 2015), or as a milder disorder, which has been called postoperative subsyndromal delirium (PSSD) (Levkoff et al., 1996; American Psychiatric Association, 2013). Both are associated with poor long-term outcomes such as longer length of hospital stay, institutionalization at discharge, and even higher mortality (Marcantonio et al., 2002; Gottesman et al., 2010; Saczynski et al., 2012; Cole et al., 2013; Shim et al., 2015). Early recognition of patients at risk of PD or PSSD is likely the most important intervention for preventing or ameliorating this condition (Aldecoa et al., 2017).

Intraoperative electroencephalogram (EEG) features have been associated with an increased risk to develop PD, like a higher suppression rate or a low bispectral index (BIS) value (Chan et al., 2013; Radtke et al., 2013; Fritz et al., 2016). However, these features depend on other factors, which can be confounders, such as anesthetic dose, older age, type of hypnotic agent, neuromuscular blockers, among others (Purdon et al., 2015a; Schuller et al., 2015; Ni et al., 2019). Other EEG patterns might be useful, such as those derived from the frequency domain (spectral) analysis. These has been used to characterize the emergence trajectories from anesthesia and the risk to develop delirium in the post anesthesia care unit (PACU-delirium) (Hesse et al., 2019). However, this analysis has not been evaluated during anesthesia maintenance to determine the risk to develop PD or PSSD. General anesthetics induce dose-dependent changes in the EEG, from low amplitude and high frequency during the conscious state to high amplitude and low frequency during anesthesia (Brown et al., 2010; Purdon et al., 2015b). Nevertheless, it is unknown whether there are intraoperative spectral differences between patients who will develop PD or PSSD and those who do not, which would allow to estimate the risk to develop an acute postoperative cognitive disorder.

The main goal of this research was to study EEG records using spectral analysis to detect potential intraoperative EEG patterns associated with PD and PSSD. A group of older patients was studied before and during the administration of inhaled anesthetics – sevoflurane or desflurane – in a real surgical scenario, using a standard 16-channel EEG. Then, PD or PSSD were diagnosed after surgery using the CAM instrument, allowing us to find an EEG pattern associated with the development of acute postoperative cognitive disorder.

## Materials and Methods

This protocol was approved by the local ethics committee at Hospital Clínico de la Universidad de Chile (Approval Number 063, September 2015). All subjects gave written informed consent before enrollment in accordance with the Helsinki Declaration. We conducted a prospective exploratory observational cohort study between May 2016 and May 2018. We included patients older than 60 years who were scheduled for elective major abdominal surgery. Exclusion criteria were history of dementia or mild cognitive impairment according to Petersen criteria (Petersen et al., 2014), encephalopathy, preoperative delirium, alcohol or drug abuse, major psychiatric disorder, and contraindication to receive inhalation anesthesia.

Physician anesthetists were allowed to provide anesthetic care according to their own clinical judgment. However, as standard care in our center, all patients were induced with 1.5–2 mg kg^–1^ propofol plus 3–4 mg kg^–1^ fentanyl or remifentanil using target-controlled infusion, according to the Minto model (Minto et al., 1997). Tracheal intubation was facilitated with 0.6 mg kg^–1^ rocuronium. Anesthesia was maintained with sevoflurane or desflurane at an age-adjusted minimum alveolar concentration (age-adjusted MAC = 1.32 × 10^–0.00303age^) (Eger, 2001) of 0.8–1.2 in an air-oxygen mixture. Ketamine, dexmedetomidine, or total intravenous anesthesia were not permitted under the research protocol; if any of these drugs were used, the patient and EEG records were excluded from the analysis. Attending anesthesiologist were blinded to EEG information. All demographic and clinical data were recorded in a database.

### Cognitive and Delirium Assessment

Before surgery, all patients were evaluated with the following cognitive and functional questionnaires: the Montreal Cognitive Assessment (MoCA) (Nasreddine et al., 2005; Delgado et al., 2017), AD8 Dementia Screening Interview (AD8) (Galvin et al., 2005), Geriatric Depression Scale (GDS) (Yesavage et al., 1982), and Pfeffer’s Functional Activities Questionnaire (FAQ) (Pfeffer et al., 1982). Preoperative delirium was ruled out applying the CAM the day before surgery. To diagnose PD or PSSD after surgery, full version of CAM was performed twice daily from the first to fifth postoperative day, by an Occupational Therapist who was blind to the EEG information. CAM-S was also applied to determine the severity of delirium episodes (Inouye et al., 2014a). The team of occupational therapist have previous experience in clinical practice and research using the CAM instrument (Tobar et al., 2010; Alvarez et al., 2017). The team is also routinely calibrated for the application of CAM by neuropsychologists, however their inter-rater reliability was not assessed.

### EEG Data Collection and Analysis

Electroencephalogram records were acquired using a 16-channel EEG (Biosemi, Amsterdam, Netherlands) placed according to the 10–20 system. The EEG signal was digitized with 24-bit resolution, at a sampling rate of 2048 Hz per channel, and referenced to the algebraic average of the left and right mastoid electrodes. For each subject, the EEG was recorded before induction of general anesthesia with eyes closed for 2 min (as a basal record) until emergence from anesthesia (intraoperative record). The analysis was repeated every 60 min for the total duration of anesthesia. Three artifact-free EEG segment of 20 s (epochs) were selected from each period to perform the spectral analysis. Epochs with noise or artifact were excluded from analysis by visual inspection. EEGLAB toolbox (Delorme and Makeig, 2004), Chronux toolbox (Bokil et al., 2010) and MATLAB (version R2017a, The MathWorks, Inc., Natick, MA, United States) custom scripts were used for EEG processing and analysis. EEG data were band-pass filtered from 0.5 to 40 Hz and down sampled to 256 Hz. Then, we computed the power spectrum for each epoch using the fast Fourier transformation function implemented in the EEGLAB toolbox (spectopo function). Next, the powers of delta (1–4 Hz), theta (5–8 Hz), and alpha (9–12 Hz) bands were obtained for each epoch and then averaged. Absolute power was expressed in a dB scale. To calculate relative power, alpha band power in μV^2^ was normalized to the total power (1–40 Hz) also in μV^2^ within the same epoch. To characterize the topography of frequency domain changes, the 16-channel data were divided into three regions of interest: frontal (Fp1 and Fp2), occipital (O1, Oz, and O2), and the average of the 16 channels (henceforth global EEG). Finally, the spectrogram was estimated using the multitaper method implemented in Chronux toolbox (window length *T* = 2 s with 0.5 s overlap, time-bandwidth product *TW* = 6, number of tapers *K* = 5 and spectral resolution of 3 Hz). Fifteen-minute electroencephalogram segments representing the maintenance phase of general anesthesia during surgery were carefully selected. The data were selected from a time period after the initial induction bolus of an intravenous hypnotic (60, 120, 180, and 240 min) and while the maintenance agent was stable defined as a stable age-adjusted MAC for at least 10 min.

### Statistical Analysis

We based our sample size calculation by a previous work (Koponen et al., 1989). They detected in patients with an acute episode of delirium that the relative power of delta, theta, and alpha were 100% different in comparison with patients without delirium. Thus, we calculated that a sample size of 36 patients would be required to detect a same magnitude of difference in any power band between patients who will develop PD/PSSD and those who did not. For this calculation, it was also considered a 20% incidence of PD/PSSD (Shim et al., 2015), 20% loss of patients, an alpha error of 0.05, and a power of 80%.

Normally distributed variables are presented as mean (SD), and non-normal variables are presented as median (IQR). The Shapiro-Wilk test was applied to evaluate normality. Student’s unpaired *t*-test and the Mann-Whitney *U*-test were used for normal and non-normal variables, respectively. Fisher’s exact test was used to compare categorical variables expressed as number (proportion). Correlation analysis was performed to estimate the strength of the relationships between variables, and receiver operating characteristic (ROC) analysis was used to determine the discriminative ability. *p*-values < 0.05 were considered significant. GraphPad Prism 7.0 (La Jolla, CA, United States) was used for statistical analyses.

## Results

### Patients Characteristics

Thirty-six patients were originally enrolled, but six patients were excluded: one patient dropped out of the study; four patients required mechanical ventilation or died during the first five postoperative days; and EEG signal quality was poor in one patient. Consequently, 30 patients were included in the final analysis (Supplementary Figure S1).

Twenty-one patients (70%) were male, and the mean age was 72.1 ± 7.0 years. Surprisingly, we found a low incidence of PD (2/30 patients, 6.7%), while another 11 patients (36.7%) presented at least one alteration in the CAM, who were considered as PSSD patients. For further analysis, both PD and PSSD (13 patients, 43.3%) were grouped and compared with patients who had a completely normal CAM (17 patients, 56.7%), henceforth control group. PD/PSSD patients were older (75.8 ± 4.9 years vs. 69.4 ± 7.2 years, *p* = 0.01) and had a lower preoperative MoCA score [25 (23–27.5) vs. 28.0 (26–28.5), *p* = 0.02] than control patients (Table 1). No differences were found in Charlson Comorbidity Index scores (Charlson et al., 1987), body mass index, education level, AD8 score, GDS, Pfeffer’s FAQ or intraoperative variables.

**TABLE 1:** Patient characteristics and physiological intraoperative variables.

**Variable**	**Control (*n* = 17)**	**PD/PSSD (*n* = 13)**	***p* value**
Sex female, n (%)	5 (29.4)	4 (30.8)	0.99
Age – years, mean (SD)	69.4 (7.2)	75.8 (4.9)	0.01
ASA, n (%)			0.52
I	5 (29.4)	2 (15.4)	
II	11 (64.7)	9 (69.2)	
III	1 (5.9)	2 (15.4)	
Charlson comorbidity index, median (IR)	5(4−6)	6(4.5−7.0)	0.31
Body mass index – Kg/m^2^, mean (SD)	25.9 (5.0)	23.5 (3.3)	0.14
Years of education, n (%)			0.93
<8 years	1 (5.9)	1 (7.7)	
8–12 years	7 (41.2)	6 (46.2)	
>12 years	9 (52.9)	6 (46.2)	
MoCA, median (IR)	28(26−28.5)	25(23−27.5)	0.02
Surgery type, n (%)			0.36
Hepatectomy	3 (17.6)	4 (30.8)	
Pancreatoduodenectomy	7 (41.2)	2 (15.4)	
Gastrectomy	4 (23.5)	3 (23.1)	
Colorectal	2 (11.8)	4 (30.8)	
Others	1 (5.9)	0 (0)	
Surgery duration – min, mean (SD)	222.6 (133.3)	223.8 (86.4)	0.98
Blood loss - mL, n (%)			0.32
0 – 499	14 (82.3)	9 (69.2)	
500 – 999	2 (11.8)	4 (30.8)	
>1000	1 (5.9)	0 (0)	
Norepinephrine infusion >0.1 mcg/kg/min, n (%)	2 (11.8)	2 (15.4)	0.99
Age-adjusted MAC at 60 min, mean (SD)	0.98 (0.16)	1.04 (0.15)	0.40
Intraoperative hemoglobin – mg/dL, mean (SD)	12.0 (1.9)	11.5 (1.3)	0.32
Intraoperative arterial pH, mean (SD)	7.34 (0.05)	7.32 (0.04)	0.35
Intraoperative glycemia – mg/dL, mean (SD)	197.5 (71.6)	182.0 (88.9)	0.74
Need of invasive mechanical ventilation, n (%)	0 (0)	1 (7.7)	0.43
Reintervention, n (%)	1 (5.9)	1 (7.7)	0.99
Length of stay >15 days, n (%)	2 (11.8)	4 (30.8)	0.36
Mortality at 90 days, n (%)	0 (0)	2 (15.4)	0.18

*Data are presented as mean (SD), median (interquartile range) or number of patients (%). PSSD, subsyndromal postoperative delirium; MoCA, Montreal cognitive assessment; MAC, minimum alveolar concentration.*

### Spectral Analysis of the EEG

**FIGURE 1:**
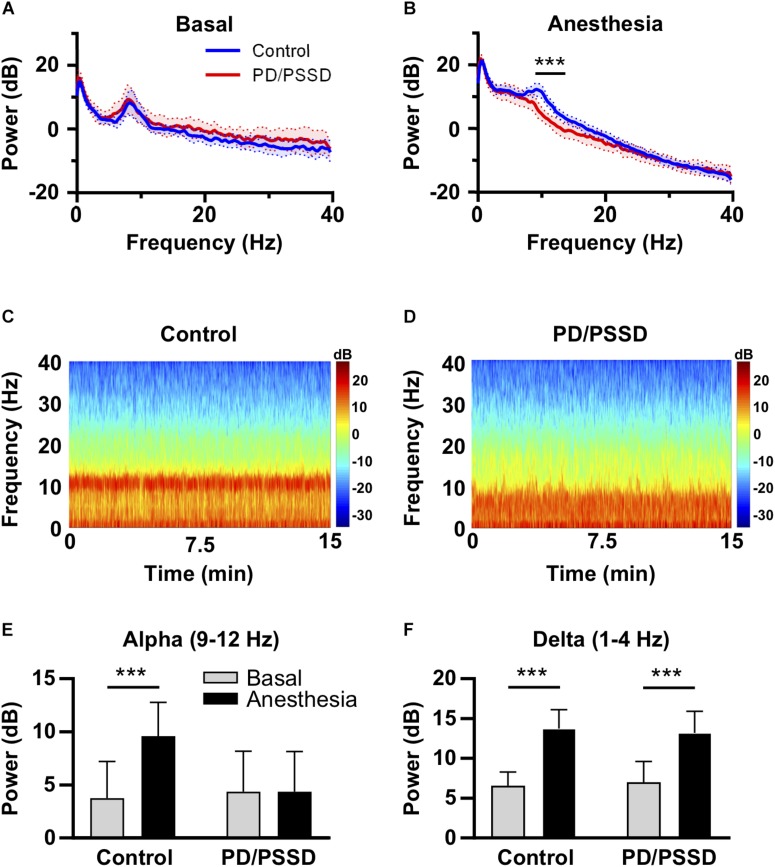
Frequency analysis from EEG records from control and PD/PSSD patients. **(A)** EEG spectrum before anesthesia. **(B)** EEG spectrum 60 min after anesthesia induction. Both spectra depict mean (thick central line) and 95% interval of confidence (colored area). Power (dB) is shown from 1 to 40 Hz. PD/PSSD patients are represented in blue and control patients in red. Black bar represent significant difference between 9 and 12.75 Hz. ^∗∗∗^*p* < 0.001. **(C)** Representative spectrogram (Fp1 electrode) of a 64-year-old control patient. **(D)** Representative spectrogram (Fp1 electrode) of a 67-year-old PSSD patient. Both spectrograms depict power according to the color scale in dB between 1 and 40 Hz frequencies and time expressed in minutes. **(E)** Absolute alpha power before (gray) and under anesthesia (black) in control and PD/PSSD patients. **(F)** Absolute delta power before (gray) and under anesthesia (black) in control and PD/PSSD patients. Each bar depicts mean and standard deviation. ^∗∗∗^*p* < 0.001.

Electroencephalogram power spectra (between 1 and 40 Hz) were calculated for each patient before anesthesia exposure with eyes closed and every 60 min after anesthesia induction. Before anesthesia, there were no differences in the spectra between patients diagnosed with PD/PSSD and control group (Figure 1A). Under anesthesia, however, patients who develop PD/PSSD had a significant lower power between 9 and 12 Hz – alpha band – in comparison with control group at 60 min after anesthesia induction (Figure 1B). This significant difference was also observed at 120, 180, and 240 min after anesthesia induction (Supplementary Figure S2). Moreover, a sustained lower alpha power also is observed in representative spectrograms from a control and a PSSD patient (Figures 1C,D). Finally, absolute alpha and delta power, both before and after anesthesia induction were calculated. The characteristic anesthesia-induced increase in alpha power was only observed in control patients and did not occur in patients who after surgery developed PD/PSSD (Control: baseline 3.8 ± 3.5 dB vs. anesthesia 9.6 ± 3.2 dB, *p* < 0.01; PD/PSSD: baseline 4.8 ± 3.9 dB vs. anesthesia 4.4 ± 3.8 dB, *p* = 0.71; Two way ANOVA; Figure 1E). On the other hand, both groups of patients showed an increase in delta absolute power after anesthesia induction (Control: baseline 6.5 ± 1.7 dB vs. anesthesia 13.7 ± 2.4 dB, *p* < 0.01; PD/PSSD: baseline 7.1 ± 2.5 dB vs. anesthesia 13.2 ± 1.8 dB, *p* < 0.01; Two way ANOVA; Figure 1F).

**FIGURE 2:**
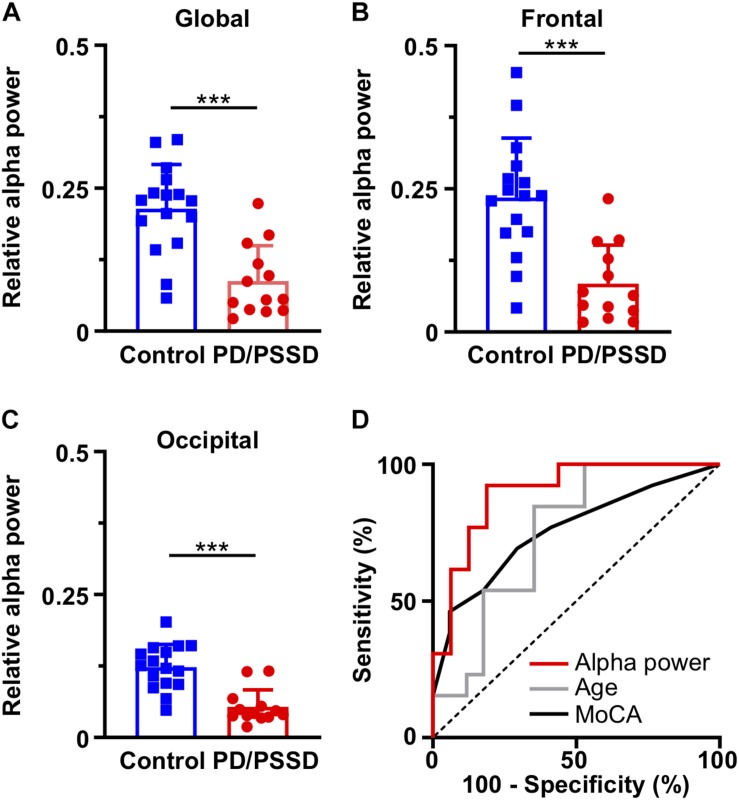
Relative alpha power in different brain region and its ability to discriminate between control and PD/PSSD patients. Relative alpha power in **(A)** global EEG, **(B)** frontal electrodes (Fp1 and Fp2), and **(C)** occipital (O1, Oz, and O2) electrodes. Control and PD/PSSD patients represented by blue and red bars, respectively. Each bar depicts mean and standard deviation. ^∗∗∗^*p* < 0.001. **(D)** ROC curves to calculate the ability to discriminate between control and PD/PSSD patients. ROC curve of alpha power is shown in red (AUC = 0.90, *p* < 0.001), of age in gray (AUC = 0.75, *p* = 0.02), and of MoCA in black (AUC = 0.76, *p* = 0.02), while dash line represents the reference line for no discrimination. *X* axis depicts 100-specificity (%) and *y* axis the sensitivity (%).

To determine whether the power spectrum differences observed were due to variations in total power or specific change in the alpha band, the relative power of alpha band was calculated. Alpha power – define as the total power between 9 and 12 Hz – was normalized to total power – define as the total power between 1 and 40 Hz. PD/PSSD patients had a significant lower relative alpha power than the control group in the global EEG (0.09 ± 0.06 vs. 0.21 ± 0.08, *p* < 0.0001, Figure 2A), frontal (0.09 ± 0.07 vs. 0.24 ± 0.1, *p* < 0.0001, Figure 2B), and occipital (0.05 ± 0.03 vs. 0.12 ± 0.04, *p* < 0.0001, Figure 2C) electrodes. These differences were not observed before anesthesia induction (data not shown). Finally, as alpha peak could be below the arbitrary frequency limit of 9 Hz, we compared the alpha peak between groups. PD/PSSD patients have a lower alpha peak (8.1 ± 0.9 Hz vs. 9.4 ± 0.2 Hz, *p* = 0.0011) with a smaller relative power (11.2 ± 4.7 vs. 15.9 ± 4.4, *p* = 0.0088) than control patients.

### ROC Analysis

Receiver operating characteristic analysis was performed to determine the ability of the relative alpha power, age and MoCA to discriminate between PD/PSSD and control patients. Global relative alpha power had an area under the curve (AUC) of 0.90 (CI 0.78–1, *p* = 0.0004) (Figure 2D), being capable to discriminate PD/POSSD patients from control patients with a sensitivity of 0.79 and a specificity of 0.86 (cut-off point = 0.13). While, age and MoCA have AUC of 0.75 and 0.76, respectively.

### Anesthetic Dose

**FIGURE 3:**
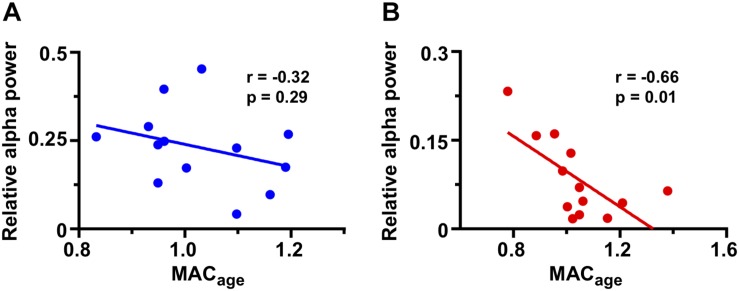
Correlation between frontal relative alpha power and age-adjusted MAC in both groups of patients. **(A)** Correlation of control patients showing in blue. **(B)** Correlation of PD/PSSD patients representing in red. Pearson coefficient, regression line, and *p*-value are shown in each graph.

As anesthetic dose is a potential confounding factor in the developing of PD/PSSD, we compared the age-adjusted MAC between the two groups. We also calculated the correlation between age-adjusted MAC and relative alpha power for PD/PSSD and control patients. First, there was no difference between age-adjusted MAC of both groups, neither at the time of EEG analysis (PS/PSSD: 1.04 ± 0.15 vs. Control: 0.98 ± 0.16, *p* = 0.4, Table 1) or in any other time during the surgery. Second, age-adjusted MAC was negatively correlated with relative alpha power in patients with PD/PSSD (*r* = −0.66, *p* = 0.01), but not in control subjects (*r* = −0.32, *p* = 0.29) (Figure 3), although this lack of statistical significance could reflect a lack of power, since this study was not designed to evaluate this relationship. Finally, other potential confounding variables, such as systolic blood pressure, diastolic blood pressure, mean blood pressure, heart rate, and peripheral oxygen saturation did not differ between the two groups of patients (Supplementary Table S1).

## Discussion

This study presents the novel finding that patients who present any postoperative alteration in the CAM (i.e., PD and PSSD) do not have the usual alpha power induced by volatile general anesthetics. Interestingly, there were no apparent differences in the spectra between groups before anesthesia induction. Therefore, PD/PSSD susceptibility is unmasked under the effects of general anesthetics, and it is manifested as a reduced intraoperative alpha power induced by anesthetics.

### Delirium and Subsyndromal Delirium

Postoperative delirium and PSSD were diagnosed using CAM questionnaire and were grouped together to be compared with patients without any alterations in this questionnaire. The rationale to include PD and PSSD in the same group was due to SSD – denominated as attenuated delirium syndrome in the DSM 5 – is considered as a milder and previous condition of delirium. There is compelling evidence that those patients with SSD cannot be considered as normal (Cole et al., 2003; Boettger et al., 2018), especially, when patients have alterations in the attention domain, which is a major diagnostic criterion for delirium (American Psychiatric Association, 2013; Meagher et al., 2014). In our 11 PSSD patients, 9 (81.8%) had alterations in the attention domain, while 6 (54.5%) had altered level of consciousness. Patients who develop PSSD have also poor long-term outcomes (Denny and Lindseth, 2017). In a critical care unit, patients with SSD had an increased hospital length of stay (Serafim et al., 2017) and institutionalization (Brummel et al., 2017). Patients with a stroke and SSD had a higher risk of poor functional outcome compared with patients with a stroke but without SSD (Klimiec-Moskal et al., 2019). After surgery, patients with SSD had a significant increase in hospital length of stay and further decline in functional status (Shim et al., 2015). Finally, patients with SSD also have an increased risk of fall (DeCrane et al., 2012) and one-year mortality (Ouimet et al., 2007; Cole et al., 2013). In conclusion, PSSD and PD correspond to a same disease with different degrees of severity and is important to differentiate patients with either, incomplete or complete diagnosis of delirium, from normal patients.

### Previous Findings in Alpha Power and Delirium

General anesthetics induce an increase in alpha power in the frontal region (Purdon et al., 2013). However, we observed that the increase in alpha power was not elicited by sevoflurane or desflurane in patients who developed PD/PSSD. This EEG phenotype unmasked by general anesthesia may be considered a potential PD/PSSD predictor. Previous studies have examined the EEG spectrum in a group of patients during an episode of delirium, who presented both, a high delta/theta and low alpha power compared with cognitively normal subjects (Koponen et al., 1989). This indicates that normal cognitive patients who did not elicit an increase in the alpha power by general anesthetics are at risk to develop PS/PSSD, which is a similar EEG pattern in patients undergoing a delirium episode. Interestingly, our subjects who develop PS/PSSD did not have any differences in the EEG spectrum before anesthesia induction compared to control patients. On the other hand, a negative correlation between alpha power and the anesthetic dose was only found in patients who developed PD/PSSD. Thus, this could indicate that in the clinical dose range of volatile anesthetics – 0.8 to 1.2 age-adjusted MAC – a dose-dependent effect in the EEG spectrum is observed only in patients at risk to develop PS/PSSD. However, this observation must be confirmed in future studies with an appropriate sample size.

### Alpha Power Mechanism

The neuronal mechanisms underlying the anesthesia-induced alpha pattern are only partially understood. Recent data suggest that anesthesia-induced alpha oscillations reflect reverberations in a thalamo-cortical loop, which in turn may be caused by hyperpolarization of the thalamus (Ching et al., 2010). Our data may be indicating that a deficit in this thalamo-cortical loop might be a predictor of a bad postoperative neurocognitive outcome. Moreover, a preoperative cognitive impairment and older age are also associated with a lower anesthesia-induced alpha power (Purdon et al., 2015a; Giattino et al., 2017). Both conditions are known risk factors to develop PD. Probably, older patients with an aging brain and/or poor preoperative cognitive performance are not able to maintain a stable thalamo-cortical synchronization during anesthesia, which is expressed as a reduced alpha power phenotype. Indeed, in our cohort of patients, we found the same correlation shown by Giattino et al. (2017). Patients with higher preoperative MoCA presented a higher intraoperative alpha relative power (*r* = 0.49, *p* = 0.005). While in other study, some patients presented a transient lost of the alpha power during maintenance of general anesthesia (Hight et al., 2019). The authors suggest that this transient loss in alpha power could be due to a noxious stimulus which might generate a depolarization in the thalamus. This observation supports the idea that alpha power induced by anesthetics depends on thalamus hyperpolarization. Nevertheless, in our study PD/PSSD patients failed to increase alpha power during anesthesia, even in the absence of surgical stimulus.

### Other EEG Features Associated With Postoperative Delirium

Intraoperative suppression rate has been associated with the development of PD in a time-dependent manner (Fritz et al., 2016). However, EEG suppression is a dose-dependent phenomenon, generally due to an inadvertent anesthetic overdose, among other causes (Ching et al., 2012). Here we used age-adjusted MAC of sevoflurane or desflurane to guide anesthesia and we did not observe any differences in anesthetic dose between the two groups. Thus, the lack of alpha power induced by anesthetics seem to be independent of anesthetic dose in terms of the ability to discriminate PD/PSSD from control patients. Second, low intraoperative BIS values have been also associated with the risk of developing PD (Chan et al., 2013; Radtke et al., 2013). However, BIS is a processed index from the frontal electroencephalographic signal (Rampil, 1998), and the algorithm to generate it is unknown. Moreover, this index has a poor performance with some drugs – dexmedetomidine and ketamine – and in older patients (Renna et al., 2003; Aime et al., 2012; Hajat et al., 2017; Ni et al., 2019; Whitlock and Sleigh, 2019). Similar to suppression rate, low BIS values could indicate either a higher anesthetic dose or increased sensitivity to the anesthetic (Sessler et al., 2012). The results of the ENGAGES trial suggest that an increased sensitivity to the anesthetics is more important than to avoid an overdose – guided by BIS – for the developing of PD (Wildes et al., 2019). Our results also show that an increased sensitivity is relevant, but only in patients who developed PD/PSSD. In these patients we observed a negative correlation between alpha power and anesthetic dose. Finally, Hesse et al. (2019) studied the association of EEG trajectories during emergence from anesthesia with PACU-delirium, and they found that an emergence without spindle-dominant slow-wave anesthesia was strongly associated with PACU-delirium. These results might be complementary to our observation because a low alpha power during maintenance of anesthesia could be associated with inadequate EEG pattern during emergence, and together constitute a high-risk EEG phenotype for patients to develop both PD/PSSD and PACU-delirium. Therefore, BIS and suppression rate have been associated with delirium. However, both have some limitations, and in a recent study, these parameters were not useful to prevent delirium when they were used to guide the dose of general anesthesia (Wildes et al., 2019). Thus, future studies in the field may evaluate whether to guide the anesthesia using the alpha power in the EEG could reduce this complication.

### Strengths and Limitations

One of the strengths is the use of a standard 16-channel EEG. Most publications have used processed EEG from frontal electrodes (e.g., BIS, SedLine) (Chan et al., 2013; Radtke et al., 2013; Fritz et al., 2016), which are easy to use in the clinical setting but the electrical information is limited. For example, electrical signal from other brain areas different from frontal regions cannot be analyzed with these monitors. In fact, the association between low alpha power and the development of PD/PSSD was stronger in occipital electrodes. Another strength is the pragmatic design, in terms that anesthesia was standard in a real surgical environment.

A limitation is the small sample size, which was useful for characterizing the EEG spectrum and for identifying a potential EEG marker of PD/PSSD, but insufficient to confirm the alpha-band power as a biomarker. However, the goal of our study was to explore -as a first step- to find a potential biomarker for predicting PD that could sustain future clinical studies. Another limitation is the low incidence of PD in our patient sample (6.7%) compare to the reported incidence, which is nearly 20% in patients with similar characteristics. In our hospital, the incidence of PD is usually low (Gutierrez et al., 2018; Tobar et al., 2018), as confirmed by twice-daily CAM evaluation by a professional team. The main factors could be the following: benzodiazepines are not used, non-pharmacological preventive measures of PD are applied during all perioperative process as a clinical standard, patients who attend our hospital have in general a high level of education, and the lack of inter-reliability was not assessed across the occupational therapists, among others. Hence, we decided to include both PD and PSSD patients, because these types of delirium may represent the same pathology at different levels of severity (Cole et al., 2013). Interestingly, a low alpha power showed a high discriminatory ability even when most patients had PSSD, thus this EEG pattern is sensible enough even to discriminate patients in the milder part of the delirium spectrum.

## Conclusion

In summary, the lack of the alpha band induced by anesthetics might be associated with an increased risk to develop PD or PSSD, independent of the anesthetic dose. Future studies approaching the underlying neuronal mechanism will shed light to understand the brain condition of patients undergoing general anesthesia. Undoubtedly, intraoperative EEG analysis in the frequency domain is a promising tool to identify patients at risk of developing poor postoperative cognitive outcomes.

## Data Availability Statement

The datasets generated for this study are available on request to the corresponding author.

## Ethics Statement

The studies involving human participants were reviewed and approved by Comité Ético-Científico o Investigación Hospital Clínico de la Universidad de Chile. The patients/participants provided their written informed consent to participate in this study.

## Author Contributions

AP: study design. RG, FR, MV, IL, and AP: patient recruitment and data collection. RG and IS: EEG recording. CB: delirium assessment. RG and JE: EEG analysis. RG, JE, MV, IL, and AP: data analysis. RG, JE, and AP: wrote the manuscript.

## Conflict of Interest

The authors declare that the research was conducted in the absence of any commercial or financial relationships that could be construed as a potential conflict of interest.
