# Supplementary material for: Intraoperative Low Alpha Power in the Electroencephalogram Is Associated With Postoperative Subsyndromal Delirium
Source: Front Syst Neurosci. 2019 Oct 18;13:56. doi: 10.3389/fnsys.2019.00056 (PMC6813625; doi:10.3389/fnsys.2019.00056)
Supplement: Supplementary file 1 [file Data_Sheet_1.docx]

Supplementary Material

| **Table S1.** Intraoperative hemodynamic and physiological variables | | | |
| --- | --- | --- | --- |
| **Variable** | **Control** | **PD/PSSD** | **p value** |
|  |  |  |  |
| Systolic blood pressure, mmHg | 113.5 (25.7) | 121.9 (27.9) | 0.47 |
| Dyastolic blood pressure, mmHg | 61.6 (16.9) | 61.7 (15.52) | 0.99 |
| Mean blood pressure, mmHg | 78.8 (17.8) | 81.8 (17.8) | 0.70 |
| Heart rate, bmp | 78.3 (15.1) | 70.3 (12.9) | 0.20 |
| Oximetry | 97.5 (2.6) | 98.8 (1.1) | 0.15 |
| Data is shown as mean and standard deviation | |  |  |
| mmHg: millimeter of mercury; bpm: beats per minute | |  |  |


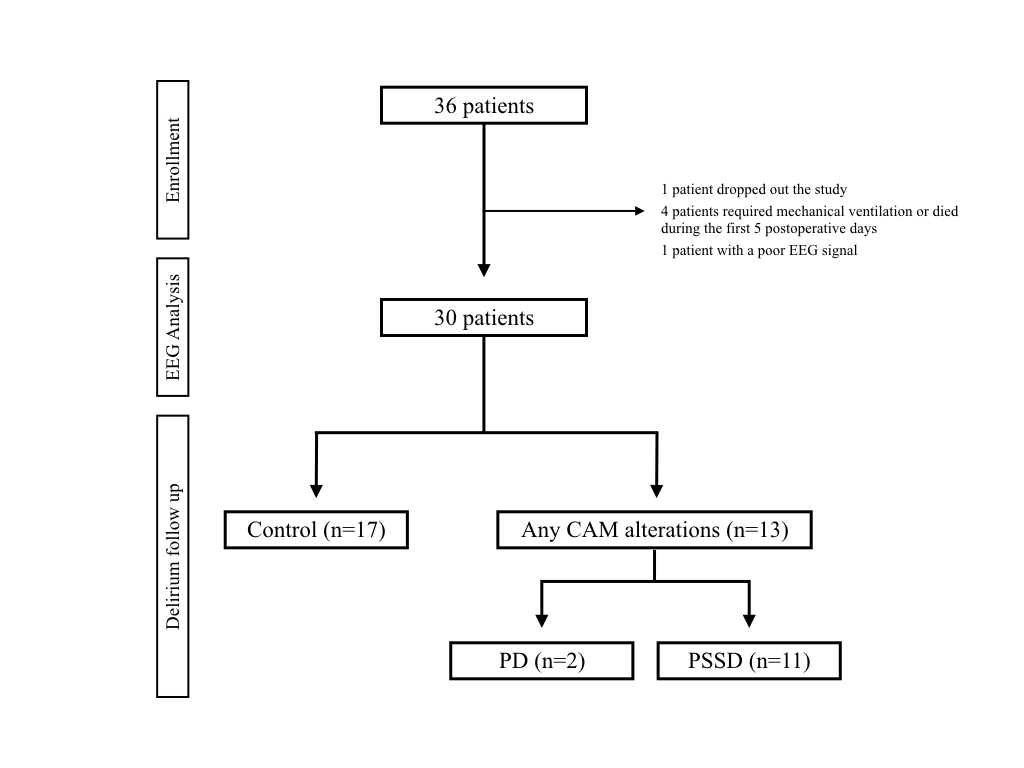


**Supplementary Figure 1.** Flow-chart of patients included in the study. EEG: electroencephalogram; CAM: Confusion Assessment Method; PD: Postoperative delirium; PSSD: Postoperative subsyndromal delirium.

**
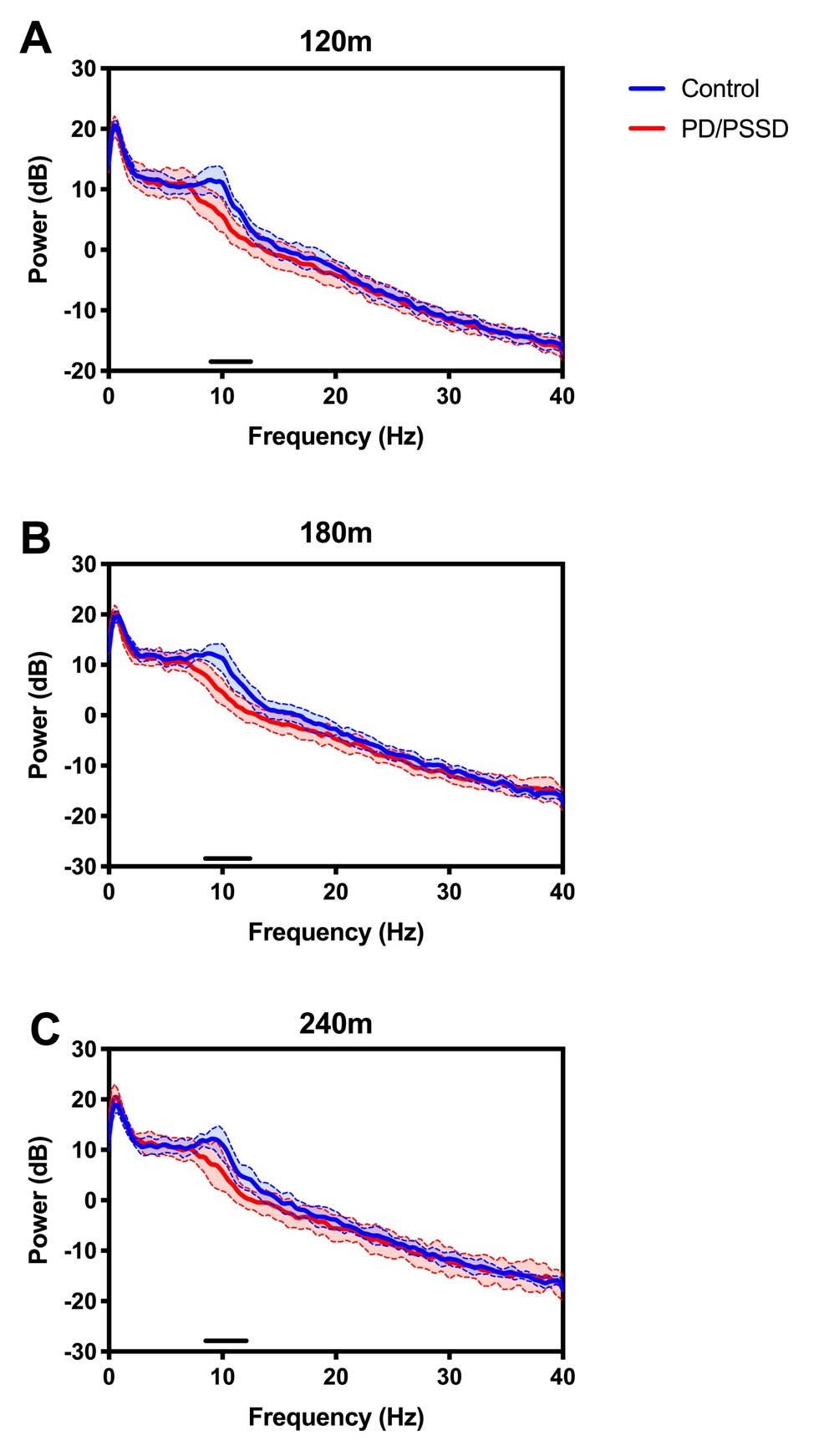
**

**Supplementary Figure 2.** Frequency analysis from EEG records of control and PD/PSSD patients. The average spectrum during anaesthesia from global EEG at 120 (A), 180 (B) and 240 (C) minutes. Spectrum represented as mean (thick central line) and 95% interval of confidence (coloured area). Power (dB) is shown from 1 to 40 Hz. Control patients are represented in blue and PD/PSSD patients in red. Each black line represents a statistical difference between the two groups (p<0.05). PD: Postoperative delirium; PSSD: Postoperative subsyndromal delirium; dB: decibels.
